# An Old-Fashioned Election—How Not to Elect a House-Surgeon

**Published:** 1895-04-20

**Authors:** 


### The Institutional Workshop.

### HOSPITAL ADMINISTRATION.

### AN OLD-FASHIONED ELECTION.—HOW NOT TO ELECT A HOUSE-SURGEON.

By Our Special Commissioner.

Many able writers have described a parliamentary election in the good old days; none, so far as we are aware, ever attempted to describe the appointment of a medical officer in a county infirmary under the old system of election which at one time prevailed throughout the country. So far as we can ascertain, there are very few places where the old system still prevails, and it was our good fortune to be present and to take part on a recent occasion in an old fashioned election of a house-surgeon to a county infirmary within a hundred miles of the metropolis. It is generally supposed that, owing to the newspaper press, the invention of the telegraph, of electricity, and of other developments, that provincial society is nowadays much broader and wider in its sympathies, and less affected by local considerations, than ever before. However true this view may be of certain neighbourhoods and places, it is certainly not invariably true, and one may occasionally meet with a locality where the conditions of society are such that the spirit of party permeates it through and through, so that the interests of one member are regarded as the interest of all, although none but the person immediately concerned may in reality have anything whatever to do with the question at issue. It is desirable to say this much by way of preface, or it may be possible that some may regard what follows as a fairy tale, without foundation or warrant. It is, in fact, a sober description of what actually took place under the circumstances already named.

#### The System and Rules.

What, then, is the old system? It provides that every subscriber of a certain sum per annum, and every donor of a certain sum, shall have the right of taking part in the election of the honorary and resident members of the medical staff, the secretary, matron, and the superintendent of nurses; that no new subscriber or donor shall have the right to vote unless their names have been entered upon the books at least one month before the date fixed for the election; that all elections shall be made by ballot if required by three governors present, and that at the ballot every governor whose subscription is paid shall have two or three votes, according to the amount subscribed; that ladies alone shall be allowed to vote by proxy, and that all other governors must attend and vote in person. In all elections where there are more than two candidates, if a majority of the whole number of votes does not appear for any one candidate upon the first scrutiny, the chairman shall proceed to a second ballot, leaving out the candidate who has the fewest votes, and so on until a majority be found. Nor is this all, for, when succeeding ballots have reduced the candidates to one, that candidate is not to be elected unless a majority of the votes of those present is first recorded in his favour. Now, the governors at a county infirmary usually number from three to eight hundred people at the least, they reside in all parts of the county, and each one has to be canvassed. Of course, under ordinary circumstances, where there are no local candidates, and no local feeling is excited, the governors are content to leave the election of their officers in the hands of the few who attend the weekly board habitually. When, however, a local candidate comes into the field, and especially when there are two local candidates, then the fat may indeed be soon in the fire. In the latter circumstances, each of the two candidates is sure to have an influential friend in the county, and that friend sets to work to whip up everyone that he can get at to vote for his man, so as to prevent the candidate of the other fellow from being elected. Thus, sometimes, if it is known that a vacancy is probable, canvassing may begin three months before any vacancy is declared, and in such cases the amount of interest excited, and the quantity of heat which may be generated, are surprising.

#### A Recent Example.

The election in which the writer took part occurred on market day in the county town at the board room of the infirmary. The system we have just described works out somewhat as follows: By the week before the election, every governor, or the majority of governors, have been canvassed by each candidate or his friends, for the last time; on the preceding day, letters and telegrams are despatched in all directions, urging the voters to come up to the ballot; and on the day itself, cabs and other vehicles are sent off to the houses of the more decrepid of the governors, with a view to tempt them to come by the offer of a free ride to the infirmary gates. It is an amusing fact, which we understand to be true, that people of eminence in the county are to be seen canvassing in all sorts of places in order to secure a few votes. The reasons which induce the voters to be present and to record their vote in favour of A or B, are founded upon every conceivable ground except the relative merits of the two competitors, one of whom has to be elected. Competition being keen, men and their wives go into two different camps at the election. Thus, the wife votes for A because her third cousin once removed is supporting him, and she feels as a matter of blood that she is bound to follow her family leader. The husband, on the other hand, votes for B because his doctor is a friend of a man who is taking an active part in B's support. In the course of the election, politics have some weight. It is understood that if the other fellow gets in, it will make the Tories unduly elated, and so several Nonconformist divines, who may have never entered the infirmary walls during the whole of the years that their congregations have subscribed to the institution, appear in force to support the Radical cause by voting for A or B, as the case may be. The town and the county talk of nothing else but this election for weeks, and a stranger ringing any bell, and asking to see the master of the house, is sure to be asked by the servant, "Is it the election, sir? And are you Mr. A or Mr. B?"

#### How the Election was Conducted.

The proceedings in the board-room were remarkable in many ways. Thus, although the room was quite

ARMY MEDICAL LIBRARY

large enough to hold the hundreds of governors who put in an appearance, there were not sufficient seats for a quarter of the number who attended. The whole preliminary proceedings were conducted in dumb show. The chairman was inarticulate, and after him came first one gentleman and then another who was supposed to propose somebody, and then after them two other gentlemen, who were supposed to propose somebody else, but not one word of any kind did anyone present hear from the lips of the five speakers who addressed them. Then there was a pause. The chairman gave the meeting no directions, but the governors on entering had noticed two small screens on either side of the room behind the chairman, which were labelled "ballot." As nobody spoke or said anything, the governors, who were most of them anxious to get out of the room as fast as they could after recording their votes, made a rush for the screens, and on getting behind them found some plain narrow wooden boxes about five feet long, a foot wide, and eighteen inches deep. Into each of these boxes was inserted four tubes like the mouth of a speaking tube, and two of them were labelled A and B respectively. The other two, though open, were not marked in any way. The governors stood and looked at these boxes, but as there were no directions and nothing of a size which would go down the tube in sight, they gave it up, turned from behind the screens, and made a rush for the board-room table. There they found two gentlemen at each end with lists, and it was ultimately discovered that anyone whose name commenced with the letters from A to L was to go to the bottom of the table; anyone coming within the letters M to Z to go to the top of the table and have his name ticked off the list. After struggling and fighting and spluttering for some thirty minutes, a persevering governor succeeded in getting to the clerk at the table, who, on hearing his name, looked at the list, and said "Two," or "Three," as the case might be, when another gentleman handed to the governor two or three marbles from a bowl of which he had charge. The governor then returned to the screen, and if he was a man of extraordinary intelligence, he dropped all the marbles he received into the mouth of the tube which was labelled with the name of his candidate. To the cautious voter, these four tubes fixed in the lid of one box caused some difficulty. Thus, one clergymen present came back with a troubled face to ask the clerk, as there were two tubes (the other two had been stopped up with paper by a considerate governor), was he to understand that he was to put one marble into each tube, or what was he to do? Some of the electors were so old that they could not see the names on the tubes, and we saw two old gentlemen hold a serious conference, and then solve the difficulty in this way. One of them approached a tube and took one of his marbles and dropped it in, and listened attentively. He did not seem satisfied, and said something to his friend, who then approached the same tube and dropped one of his marbles in, also listening intently. He smiled, looked up, and said "That's all right," dropped in his other marble, and so did his friend. We found that their idea was to select a tube which other governors had put marbles down. They argued that if they listened intently they could ascertain whether their marble fell upon wood, or whether it struck another marble. If it struck another marble it was all right, because they were doing as others had done before them, but if they struck wood they must try another tube.

The plan of campaign adopted by the governors was most effective. You were suddenly startled by hearing the tramp of a number of men, and you found that a battalion of six or eight governors was being marched up with their doctor at the head, and a representative of the candidate for whom they were going to vote at the tail. They went straight into the room, got their marbles and went to the ballot box. This dodge seemed to have great weight with some governors who had not made up their minds. It was at least imposing, and we suppose to a certain extent effective too. The majority of governors left the board-room directly they had voted. One party who we understood had come from a distant part of the county, and had risen very early in the morning, on arrival took up their position in a corner and proceeded to at once open some paper parcels they had with them and devour bread and cheese with great gusto. When the voting was at length completed the ballot boxes were brought to the table, and a gentleman was told off to count the number of marbles which each candidate had received. The plan of counting was original, but highly dangerous to accuracy. Ten balls were taken out, duly counted, and put into a bowl by the side of the counter, who then said "One." Ten more balls were then taken out in the same way, and put into the bowl, with the declaration "two," and so on. When the counter got to "four" or "five," on taking out the sixth batch of balls he became rather doubtful as to whether it was forty or sixty, and whichever way he decided in his own mind he might be wrong, but there was no one to check him and no one to know. But that was, after all, a small matter, as it was done in perfect good faith and temper. After the boxes containing the marbles put in by the governors had been counted, a third box was produced, containing the proxies. The proxy papers had been examined, and it was found that, with an impartiality which was in all respects admirable, several ladies had voted for both candidates. This caused no difficulty, however, as it was agreed that each candidate should have the full benefit of all the proxies he produced, no matter whether they were duplicates or not.

Just as the examination of the proxies was completed there was a slight commotion outside, a sound of horses' hoofs on the gravel, a foaming steed was pulled sharply up at the door, and in rushed a gentleman with another proxy paper, which was duly examined and admitted. In the end some six hundred votes were recorded, and the successful candidate had a majority of twelve; that is, of six governors out of about some three hundred who had recorded their votes. One of the most curious things about this ballot was the fact that it was no ballot at all. Anyone was at liberty to stand against the wall behind the ballot box, and to see how everybody voted. Again, no scrutiny was demanded, no check was kept as to the ticking of the governors' list, the authenticity of the proxies, or the accuracy of the counting, and in the absence of a scrutiny, we have very little doubt that had it taken place it is just as likely that the unsuccessful candidate would have been elected as that the successful candidate would have been found to have received twenty more votes than those which were credited to his account. Thus ended one of the most amusing, and in some senses one of the most pathetic scenes at which we have ever officiated in connection with the voluntary hospital system of this country. We are happy to be in a position to express some confidence that so far as the particular institution is concerned at which these proceedings took place, steps will be taken without delay to alter the system, and thus prevent the repetition of proceedings which are unworthy of a great medical institution with a splendid record of good work well done for a large population in an English county. We were told on the authority of the secretary that the proceedings here recorded were quite tame compared with those which took place thirty years ago, on the occasion of the last contested election when local feeling ran high. Then a matron had to be elected, and the balloting was persisted in until four o'clock in the afternoon, the excitement being intense all the time. If there had been half a dozen candidates to select from on the occasion we refer to, our belief is that the proceedings might have gone on for twenty-four hours, if they had not terminated earlier in riot and confusion.

### The Right System.

The least objectionable and most satisfactory system for the election of the officers of a hospital is by a committee of election. The power to elect is vested in a special committee called the committee of election, consisting of (*a*) the committee of the hospital as defined by the laws; (*b*) the honorary physicians and surgeons; (*c*) such members of the governors chosen by ballot at the meeting of the committee called or made specially for the purpose, at which a vacancy is reported, as, together with (*a*) and (*b*), will make up the number of the committee of election to one hundred. At the meeting at which the ballot takes place, advertisements for candidates for the vacant post are ordered to be inserted in the newspapers. The applications when received are handed on to the medical committee, who report to the chairman of the committee of election whether the candidates are duly qualified according to the laws. Immediately after the ballot the secretary issues a notice to every member of the committee of election of the time and place of the election and gives to every candidate simultaneously a similar notice, together with a printed list of the members of the committee of election with their addresses. If the majority of the committee of election is of opinion that it is inexpedient to elect any of the candidates, the proceedings for filling the vacancy begin again *de novo*. It will be observed that this system gives every candidate an equal chance. No canvassing can be commenced until the election committee has been selected by ballot, and any previous canvassing of the members who come under (*a*) and (*b*) disqualifies altogether. The practice is to have three or four clerks, according to the number of candidates present at the ballot, who take down the names and addresses of the governors as they are drawn from the ballot box. In this way a sufficient number of lists of the election committee are ready at the conclusion of the ballot, and every candidate can be supplied with one then. We ought, perhaps, to explain that the above system provides that the whole of the governors' names shall be placed in the ballot box, that every time there is a vacancy a fresh committee of election shall be appointed by ballot, and that in this way every governor has an equal chance of being selected to dispense the patronage of the hospital, whilst he enjoys the added interest of the uncertainty and competition entailed by the ballot. This system has the additional advantage that it entails little or no cost upon either candidates or institution, and is so found to give unbounded satisfaction to all parties concerned. In these circumstances we commend its adoption to the governors of every institution where a less simple and satisfactory plan is at present in force.

